# The Pathogenicity and Transcriptome Analysis of Methicillin-Resistant* Staphylococcus aureus* in Response to Water Extract of* Galla chinensis*

**DOI:** 10.1155/2019/3276156

**Published:** 2019-07-16

**Authors:** Shizhou Wu, Yunjie Liu, Hui Zhang, Lei Lei

**Affiliations:** ^1^Department of Orthopedics, West China Hospital, Sichuan University, Chengdu, China; ^2^State Key Laboratory of Oral Diseases, Department of Preventive Dentistry, West China Hospital of Stomatology, Sichuan University, Chengdu, China; ^3^West China School of Public Health, Sichuan University, Chengdu, China

## Abstract

**Aim:**

Antibiotic abuse contributes to the emergence of methicillin-resistant* Staphylococcus aureus* (MRSA). It is increasingly important to screen new antimicrobial agents for the management of MRSA infections.* G. chinensis, *a nontoxic Chinese herbal medicine, is considered a potential antibacterial agent. The aim of this study was to investigate the bactericidal effects of the aqueous extracts of* G. chinensis* on MRSA. The potential mechanisms of* G. chinensis* aqueous extract inhibition of the pathogenicity of MRSA* in vivo* are also discussed.

**Methods:**

* G. chinensis* aqueous extract was prepared and its antimicrobial activities were examined by determining its minimum inhibitory concentration (MIC). Biofilm biomass was determined by scanning electron microscopy (SEM) and confocal laser scanning microscopy (CLSM). RNA sequencing (RNA-seq) was used to evaluate differentially expressed functional pathways in MRSA treated with* G. chinensis* aqueous extract. We validated the role of* G. chinensis* aqueous extract in the invasive ability and pathogenicity of MRSA* in vivo* using a rat infectious model.

**Results:**

The results indicated that MRSA was sensitive to the* G. chinensis* aqueous extracts at concentration of 31.25*μ*g/mL.* G. chinensis* extract led to a reduction in dextran-dependent aggregation and biofilm formation in MRSA. Based on the transcriptome analysis,* G. chinensis* aqueous extracts significantly downregulated the gene expression related to biofilm formation and carbohydrate metabolism.* G. chinensis* aqueous extract inhibited the invasive ability and the pathogenicity of MRSA* in vivo*.

**Conclusion:**

The antimicrobial properties of* G. chinensis* aqueous extract are likely related to its modulation of MRSA biofilm formation and carbohydrate metabolism.* G. chinensis* aqueous extract is a promising supplementary therapy to lessen or eliminate the use of antibiotics and is a potential tool for the management of MRSA infections.

## 1. Introduction

*Staphylococcus aureus* (*S. aureus*) is a common bacterial genus in human caused infectious diseases from innocuous commensal to fatal infections [1]. As a type of gram-positive cocci and coagulase-positive coccoid bacterium of the Firmicutes phylum,* S. aureus* is carried by approximately 20%–30% of healthy humans [2]. With the acquisition of the gene* mecA*, the methicillin-resistant* Staphylococcus aureus* (MRSA) is emerged constitutively. The* mec* operon is carried by the Staphylococcal cassette chromosome* mec *and mostly originated from horizontal transfer from coagulase-negative* Staphylococcal* species [3].

Microbial biofilm cells show significantly less susceptibility to antimicrobial agents than planktonic cells [4]. Correspondingly, more biofilm is identified in MRSA strains compared with methicillin-sensitive* S. aureus* strains [5]. Polysaccharide intercellular adhesion (PIA) is a vital component necessary for the biofilm organization of* S. aureus* [6]. The poly-N-acetyl glucosamine (PNAG) polysaccharide deposited on the surface of the cell wall, also referred to as PIA, is synthesized by glycosyl transferase enzymes that are encoded by the* ica* operon [7].

**Figure 1:**
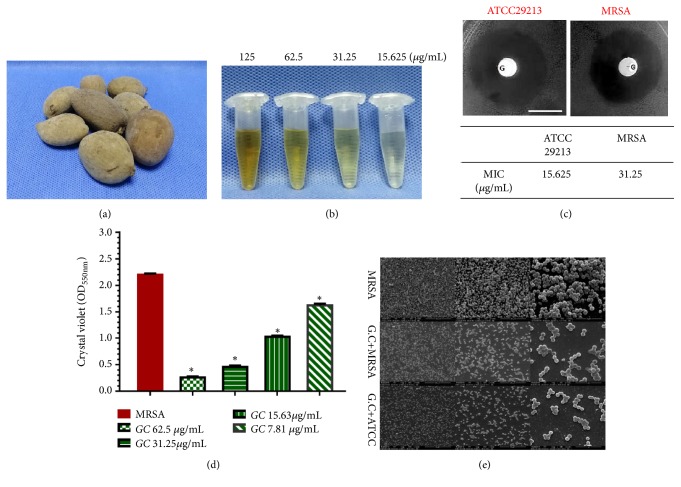
*Sensitivity of S. aureus to G. chinensis extract.* (a) Morphology of dried* Galla chinensis*; (b) morphology of* G. chinensis* extract solutions; (c) the inhibition zone (the upper lane) and the minimum inhibitory concentration values (the lower lane) of* G. chinensis* extract solution for* S. aureus*; (d) crystal violet stain for methicillin-resistant* S. aureus* cells treated with different concentrations of* G. chinensis *extract for biomass comparison (*∗*p<0.05, n=10); (e) scanning electron microscopy (SEM) observation of the biofilm architecture of MRSA strain, MRSA treated with* G. chinensis *(1/2 MIC), and* S. aureus* ATCC with* G. chinensis* (1/2 MIC), respectively, at 24 h of growth. The MRSA biofilm treated with* G. chinensis *(1/2 MIC) presented a reduction in the extracellular matrices and only small microcolonies existing compared with untreated MRSA strains.

As the MRSA strains have been reported to be resistant to conventional antibiotics, new antimicrobial agents are urgently needed for the management of MRSA infections. Recently, it has become increasingly important to screen effective Chinese traditional medicines as potential sources of drugs for the management of* Staphylococcus* drug-resistance [8].* Galla chinensis* (*G. chinensis*), a nontoxic Chinese herbal medicine, is naturally formed when* Rhus chinensis* Mill is parasitized by* Melaphis chinensis* Bell.* G. chinensis* is considered to be a potential antibacterial agent [9, 10]. Gallnuts are a group of very special natural plant-insect symbiont products measuring 2.5–9 cm in length and 1.5–4 cm in diameter as shown in Figure 1(a) [11, 12]. The compounds and extracts from* G. chinensis* are rich in gallic acid, gallotannin, and hydrolysable tannins and possess antimicrobial characteristics [10, 11, 13]. Furthermore, safety evaluation tests have shown that little acute or chronic toxicity is present when* G. chinensis *extracts are taken at lower doses [14].

Previous investigations have demonstrated that components or extracts from* G. chinensis* have bactericidal activities against* S. aureus,* including growth-inhibitory and biofilm-reducing effects [9, 13]. However, our understanding of the antimicrobial characteristics of* G. chinensis* water extract to MRSA is still very limited. Taken together, the aims of this study were as follows: (1) to determine whether the aqueous extract of* G. chinensis* has* an antibacterial* effect on MRSA, (2) to perform transcriptome analysis of MRSA in response to treatment with* G. chinensis* aqueous extracts, and (3) to determine whether the aqueous extract of* G. chinensis* inhibited the pathogenicity of MRSA* in vivo*.

## 2. Materials and Methods

### 2.1. Preparation of an Aqueous Extract of G. chinensis

The fresh* G. chinensis* was harvested in autumn and placed in boiling water until the surface turned gray. Then, the gray* Galla* was dried in the air after removal of the larvae [11]. We purchased the* G. chinensis* from Zhewan Traditional Chinese Medicine of Limited by Share Ltd. (*Galla chinensis*, Certificate No. AH20150176). The obtained* G. chinensis* aqueous extract was processed as previously described [15, 16]. Briefly, the obtained* G. chinensis* was dried, powdered, and extracted with distilled water [16]. The solutions were concentrated using a vacuum falling filter evaporator (Iwai Co., Japan). The dried extract was dissolved in water to achieve a concentration of 10 g/L (W: V), then sterilized with a 0.2 *μ*m syringe filter, and kept at 4°C for further experiments.

### 2.2. Bacterial Strains and Growth Conditions

*S. aureus *strains of ATCC 29213 (methicillin-sensitive* S. aureus* strain) and clinical isolated MRSA strains were obtained from the Department of Laboratory Medicine (West China Hospital, Sichuan University, Chengdu, China) and cultured on conventional Baird-Parker (BP) agar plate. The pure growth of single clones was achieved and Gram's staining was conducted for initial* Staphylococcus* strains identification [5]. For incubation,* S. aureus *strains were grown in Luria–Bertani (LB) liquid (Oxoid, Basingstoke, United Kingdom) at 37°C overnight. Then, the bacterial suspensions were cultured to mid-logarithmic growth phase (optical density at 600 nm of 0.5) in LB medium for further experiments.

### 2.3. Testing Planktonic Antimicrobial Susceptibility

The minimum inhibitory concentration (MIC) test was performed in LB medium via broth microdilution techniques in the presence of approximately 1 × 10^7^ CFU/mL of* S. aureus *ATCC 29213 and MRSA strains. The LB medium contained serially diluted* G. chinensis* water extracts ranging from 3.9 *μ*g/mL to 125 *μ*g/mL. The MICs, the minimal inhibitory concentration, were defined as the concentration at which no visible turbid bacterial growth was observed. The determined MIC values were compiled for further investigations.

### 2.4. Biofilm Assays In Vitro

Biofilms were established after 24 h at 37°C in LB, GC (1/2 MIC) + LB media [17]. A crystal violet microtiter assay was used to quantitatively measure the biofilm biomass. Briefly, the biofilms were washed in phosphate buffered saline (PBS), dyed with crystal violet solutions, and then solubilized with the destaining solutions as previously described [5]. Then, the destaining solutions were transferred into a clean 96-well plate, and the optical density was measured at 600 nm [5].

The biofilm samples were washed twice in PBS and imaged with a scanning electron microscope (SEM, Inspect Hillsboro) following our previous procedures [5, 18]. The samples were serially dehydrated with ethanol solutions (30%, 50%, 70%, 95%, and 100%), dried in air, and coated with gold for imaging. Three randomly selected areas from each sample were imaged by SEM.

The anteromedial tibia cortex of the healthy rat was prepared and sliced into 4 × 4 mm specimens using a hard-tissue cutting machine (Buehler, Chicago, IL, USA). The bone samples were washed ultrasonically in distilled water for 10 min and stored in 10 mM PBS (pH 7.0) at 4°C. Then, the specimens were incubated with the MRSA strains,* G. chinensis* (1/2 MIC) + MRSA strains, and* G. chinensis* (1/2 MIC) + ATCC strains. After 24 h of coculture at 37°C, bone specimens were rinsed twice with PBS to remove the supernatants. The EPS matrix of* S. aureus* biofilms was stained with Alexa 647-labeled dextran conjugate (Invitrogen, Eugene, OR, USA) [5]. The bacterial cells in the biofilm were labeled with SYTO9 (Invitrogen, Carlsbad, CA, USA). Confocal laser scanning microscopy (CLSM, TSP SP2; Leica, Solms, Germany) was then performed [18]. For the fluorescence microscopy, the live and dead cells in the biofilms grown on the glass coverslips were distinguished with LIVE/DEAD BacLight™ Bacterial Viability Kit reagent (Invitrogen, Waltham, MA) and labeled in accordance with the manufacturer's instructions to assess the proportion of vital bacteria [18].

### 2.5. RNA Extraction and RNA Sequencing Performance

Bacterial total RNAs were extracted from mid-exponential phased planktonic methicillin-resistant* S. aureus* or the* G. chinensis* extract (1/2 MIC)*-*treated MRSA strain using the MasterPure™ RNA purification Kit (Epicenter Technologies, Epicenter, Madison, WI, USA) and purified with DNase I (Ambion) following the manufacturer's instructions. RNA quality and purity were analyzed by an Agilent 2100 bioanalyzer (Agilent Technologies). All RNA was determined to have an RNA integrity number (RIN) of 9.0 and above. Removal of rRNA was performed using a Ribo-Zero™ rRNA Removal Kit for gram-positive bacteria (Epicenter) in accordance with the supplier's specifications. The final quality and purity of the enriched bacterial mRNA were analyzed using an Agilent Bioanalyzer (Agilent Technologies) [19]. From enriched mRNAs, cDNA libraries were processed using a TruSeq™ RNA sample preparation kit (Illumina). Subsequently, RNA sequencing (RNA-Seq) was performed on a HiSeq 4000 (2x150 bp read length) at Majorbio Biotechnology Research (Shanghai, China). A Galaxy server was used to perform read mapping procedures with Bowtie 2 for Illumina [8].

### 2.6. Statistical Analysis of RNA Sequencing Data and Data Validation

Reads were mapped to the genome of methicillin-resistant* S. aureus*. Fold changes and significant differences in gene expression were calculated using edgeR (http://www.bioconductor.org/packages/2.12/bioc/html/edgeR.html) [20]. Significant differences in genes were defined as a fold-change > 2 and a threshold false discovery rate (FDR) of ≤0.05. The pathways were assigned by Gene ontology (GO) terms using Blast2GO [21]. Relative enrichment of GO terms compared with a background of GO terms was assessed using Fisher's exact tests. After combining all evidence from the gene expression level data, pathway enrichment analysis was performed, and the FDR procedure was used to correct for multiple hypothesis testing (FDR < 0.05). For the RNA-Seq data validation, quantitative real-time PCR assays were conducted to measure the expression levels of the genes (primers listed in Table A1). Briefly, total RNAs were isolated from cells harvested at mid-logarithmic growth phase and purified using the MasterPure™ RNA purification Kit (Epicenter Technologies, Epicenter, Madison, WI, USA) [5]. Contaminating genomic DNA was removed using Turbo RNase-free DNase I (Ambion). Any residual genomic DNA contamination was assessed and the quality of the RNA was determined. The reverse transcriptional reactions were processed using the RevertAid First Strand cDNA Synthesis Kit (Thermo Scientific) as previously described [5, 22].

### 2.7. Osteomyelitis Animal Model Construction and Micro-CT Imaging

Approved by the Institutional Animal Welfare Committee (West China Hospital, China, Approval No. 2018039A), 10 female Sprague-Dawley rats (260–280 g) were used in this study. Research was conducted in accordance with the nationally accepted principles for laboratory animal experiments. All animals were anesthetized by ketamine (60 *μ*g/g) and xylazine (6 *μ*g/g). Then, the right hind legs were shaved and disinfected with polyvinyl pyrrolidine-iodine. The anteromedial tibia cortex was exposed by incisions 1 cm in length, and a hole of 0.1 cm diameter was prepared on the medullary cavity as previously described [23]. The* S. aureus *clinical isolated MRSA strain was cultured on conventional Baird-Parker (BP) agar plate. The pure growth of single clones was achieved and Gram's staining was conducted for initial* Staphylococcus* strains identification. For incubation, MRSA strains were grown in Luria-Bertani (LB) liquid (Oxoid, Basingstoke, United Kingdom) at 37°C overnight. The untreated MRSA suspensions were cultured to mid-logarithmic growth phase (optical density at 600 nm of 0.5) in LB medium. The treated MRSA suspensions were grown to mid-logarithmic growth phase (optical density at 600 nm of 0.5) in LB medium and then cocultured with GC (1/2 MIC) for further experiments. For the bacterial injection, ten animals were divided into two groups, including the untreated group (n=5) injected with 100 *μ*L of LB culture medium containing mid-exponential phase methicillin-resistant* S. aureus *only (1 × 10^7^ CFU/mL) and the treated group (n=5) that was inoculated with 100 *μ*L of a mixture of mid-exponential phase methicillin-resistant* S. aureus *suspension (1 × 10^7^ CFU/mL) in LB culture medium cocultured with GC (1/2 MIC).

After suturing, all animals were observed for 4 weeks. To evaluate the infective tibias in rats, micro-CT analysis was performed using a Quantum GX Micro-CT System (PerkinElmer, Waltham, MA) as previously described [24]. The scanning conditions were as follows: kV = 90; CT *μ*A = 72; 360° scan time = 8 sec [25]. The three-dimensional images were reconstructed using Analyze 12.0 (PerkinElmer). The ratios of BV/TV (trabecular and cortical bone volume (BV) per total volume (TV)) and cortical bone thickness (Ct. Th) were analyzed. Then, we split the rat tibia shaft longitudinally for the histological evaluations. Briefly, the tibias were fixed in 10% neutral buffered formalin, decalcified in 10% EDTA, and embedded in paraffin as previously described [26]. The 5 *μ*m slices were Gram-stained to assess bacterial colonization.

### 2.8. Data Analysis

The homogeneity of data variances was assessed by Bartlett's test and the normal distribution of data was determined by the Shapiro-Wilk test. For parametric testing, the one-way analysis of variance model was used to compare the data followed by pairwise multiple comparisons.

## 3. Results

### 3.1. Sensitivity of Methicillin-Resistant* S. aureus *to* G. chinensis* Extracts

The MIC values of ATCC 29213 and methicillin-resistant* S. aureus *for* G. chinensis *aqueous extracts were 15.625 *μ*g/mL and 31.25 *μ*g/mL, respectively (Vancomycin as the positive control in Table A2). The methicillin-resistant* S. aureus* and ATCC 29213 diameters of the inhibition zones around* G. chinensis *(1 *μ*g) disks were 20.2 ± 0.5 mm and 23.3 ± 0.3 mm (n=10, P<0.05, Figure 1(c)).

### 3.2. *G. chinensis* Suppressed Biofilm and Extracellular Matrix Formation of* S. aureus*

**Figure 2:**
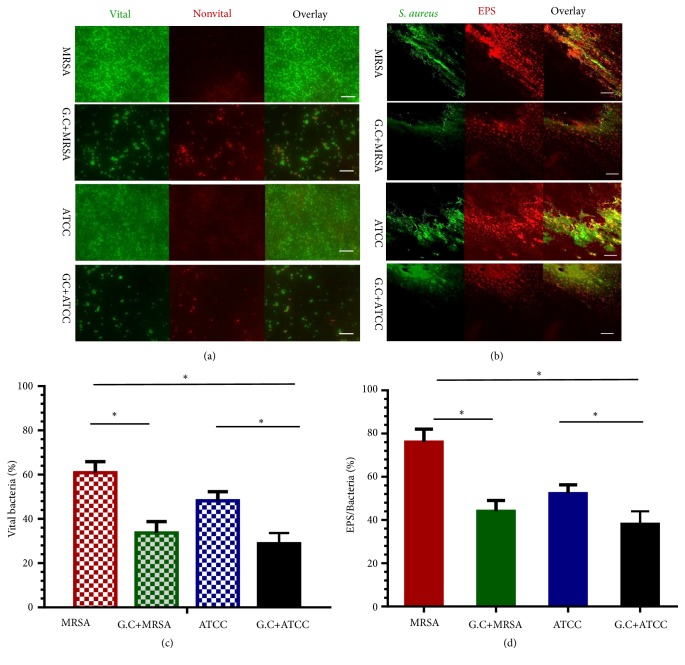
*G. chinensis extract suppressed biofilm formation and extracellular matrix of S. aureus*. (a) Double labeling of* S. aureus* biofilm. Green, viable* S. aureus* bacteria (SYTO 9); red, dead* S. aureus* bacteria (PI); scale bars, 100 *μ*m; (b) double labeling of* S. aureus* biofilm formation on bone specimens. Green, total* S. aureus* bacteria (SYTO 9); red EPS (Alexa Fluor 647); scale bars, 100 *μ*m; (c) percentage (%) of viable* S. aureus* cells in biofilm (n=10, *∗P*<0.05); (d) volumetric ratio of the EPS matrix to the bacterial biomass in the biofilms of* S. aureus* strains (*∗P*<0.05, n=10).

Crystal violet microtiter assay results revealed that biofilms of methicillin-resistant* S. aureus* treated with different concentrations of* G. chinensis* water solutions (62.5*μ*g/mL 31.25 *μ*g/mL, 15.625 *μ*g/mL, and 7.81 *μ*g/mL) were significantly impaired when compared with the control group (n=10,* P*<0.05, Figures 1(b) and 1(d)). SEM observation demonstrated that methicillin-resistant* S. aureus* cells were densely packed with extracellular matrix, whereas the methicillin-resistant* S. aureus *and ATCC strains treated with* G. chinensis* (1/2 MIC) showed reduced extracellular matrices in the biofilms interspersed among the “blank” areas, and only small microcolonies were randomly observed (Figure 1(e)). After incubation in LB medium with* G. chinensis* (1/2 MIC), both viable bacteria ratios of methicillin-resistant* S. aureus *and ATCC strains biofilms were observed by CLSM (Figure 2(a)). The proportion of viable methicillin-resistant* S. aureus* cells in the* G. chinensis *extract-treated methicillin-resistant* S. aureus* strains (33.6 ± 5.2%) was lower than the MRSA strains without intervention (60.89 ± 5.0%) (P<0.05, n=10, Figure 2(c)), which was similar to the ATCC 29213 strain treated by* G. chinensis *extract (28.8 ± 4.8%). By double-staining and CLSM observation, we found that EPS production in rat bone specimens clearly decreased in the* G. chinensis*-treated MRSA and ATCC groups (Figure 2(b)). These findings were further confirmed by quantitative data revealing that* G. chinensis*-treated MRSA cells exhibited a lower EPS/bacterial biomass (44 ± 5%) volume ratio than not-treated MRSA cells (76 ± 6%, P<0.05, n=10, Figure 2(d)).

### 3.3. Transcriptome Analysis Revealed that G. chinensis Modulates Carbohydrate Metabolism

**Figure 3:**
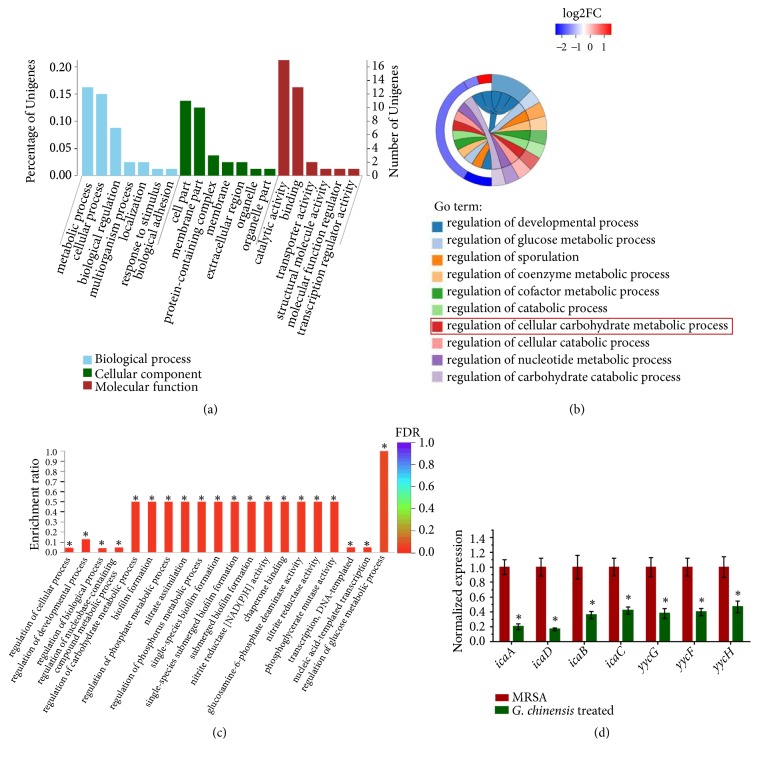
*Transcriptome analysis revealed that G. chinensis modulates carbohydrate metabolism.* (a) Gene ontology terms annotation statistics; (b) Gene ontology enrichment analysis string diagrams; the majority of differentially regulated genes were related to carbohydrate metabolic processes, shown in red; (c) significant terms in Gene ontology enrichment analysis (*∗*FDR<0.05); (d) quantitative real-time PCR (qRT-PCR) validation for the expression changes of selected genes (*∗P*<0.05, n=10).

Using RNA-Seq,* G. chinensis *extract (1/2 MIC) treatment differentially regulated genes related to the regulation of carbohydrate metabolic processes, including glucose metabolic processes and biofilm formation processes (Figures 3(b) and 3(c)). GO enrichment showed altered carbohydrate metabolic processes and biofilm formation processes, suggesting that* G. chinensis* extract affected carbohydrate utilization by methicillin-resistant* S. aureus*. We next validated the gene expression of biofilm-associated genes by RT-qPCR (Figure 3(d)). In the* G. chinensis*-treated group, the mRNA expression levels of* yycG*,* yycF, yycF*,* icaA*,* icaB*, and* icaD* were significantly lower than those in the untreated group. In particular, the expression levels of* icaA* and* icaD* in the* G. chinensis*-treated group were five times lower than those in the untreated group. Consistently, these results demonstrated that* G. chinensis* suppressed the expression of* S. aureus* biofilm-associated genes and exopolysaccharide synthesis genes.

### 3.4. Inhibition Effect of G. chinensis on the Pathogenicity of S. aureus-Infected Osseous Tissue

**Figure 4:**
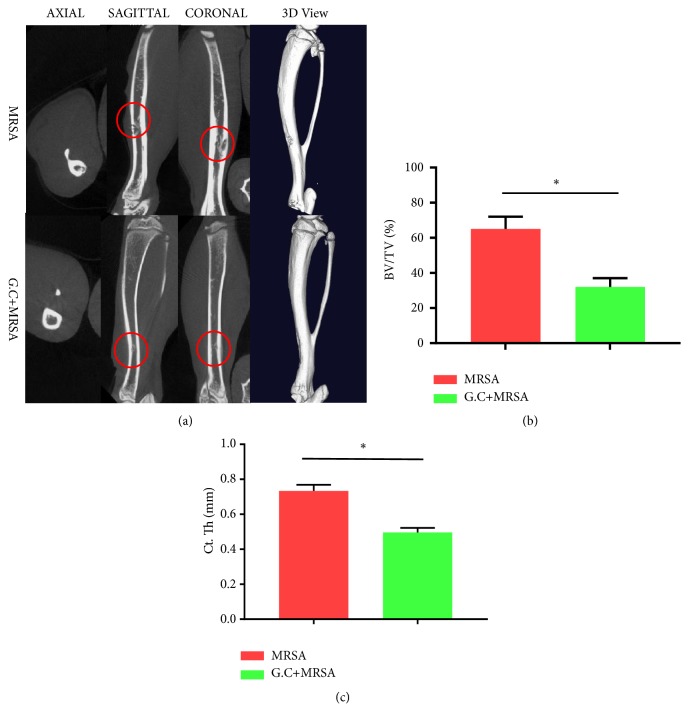
*Inhibition effect of G. chinensis on pathogenicity of methicillin-resistant S. aureus in vivo.* (a) Micro-CT assessments and 3D images of rat tibias infected by methicillin-resistant* S. aureus*; the osteomyelitis caused by methicillin-resistant* S. aureus* and the infected regions are indicated (red circle); (b) the average BV/TV values in the methicillin-resistant* S. aureus *and* G. chinensis* extract-treated groups (*∗*P<0.05, n=10); (c) the average cortical bone thickness (Ct. Th) in the methicillin-resistant* S. aureus *and* G. chinensis* extract-treated groups (*∗*P<0.05, n=10).

The micro-CT analysis showed significant osteolysis in the cortex and the thickness of the cortex was obviously increased in the methicillin-resistant* S. aureus*-infected group compared with the* G. chinensis *extract-treated group (Figure 4(a), upper lane). However, little reactive bone around the cortex was defined in the* G. chinensis *extract-treated group. This trend indicates that the* G. chinensis*-treated strains presented a limited capability to induce an infarct in infected bone tissues (Figure 4(a), lower lane). The quantitative data revealed that the average BV/TV value was 32 ± 5.2% in the* G. chinensis* extract-treated group, which was significantly lower than that in the methicillin-resistant* S. aureus* group, with a BV/TV value of 65 ± 7.1% (n=5, P<0.05, Figure 4(b)). Furthermore, the average value of cortical bone thickness (Ct. Th) was 0.73 ± 0.04 mm in the MRSA group, which was elevated compared with that in the* G. chinensis* group (0.49 ± 0.02 mm, n=5, P<0.05, Figure 4(c)). These data indicated that the* G. chinensis* extracts suppressed the pathogenesis of MRSA in a rat osteomyelitis model.

## 4. Discussion

MRSA remains among the group of high-priority multidrug-resistant organisms that requires renewed efforts for the development of new antibiotics and innovative preventive approaches [27]. Conventional antibiotics may not be effective against the bacteria that develop resistance [28, 29]. Therefore, screening for Chinese herbal medicines that lessen the use of antibiotics may be a useful method for identifying compounds suitable for infection management.* G. chinensis* contains large amounts of hydrolysable tannins, which contribute to its effective and broad activities as a topical antibacterial agent [10]. According to Buziashvili et al., the main ingredients of* G. chinensis* are gallic acid (nearly 20%) and methyl gallate (7%) [30]. It was also reported that methyl gallate and gallic acid have significant growth-inhibitory activity towards the glucosyltransferase enzymes and biofilm formation [31]. On the other hand,* G. chinensis* contains large amounts of hydrolysable tannins with higher molecular weights, which contribute to its effective and broad activities as a topical antibacterial agent [10]. The components of gallotannins along with tannins make* G. chinensis *very useful in bacterial control as these effects of tannins may be precipitated by their binding to bacterial proteins [32]. The crude aqueous extracts of* G. chinensis* were characterized previously by high performance liquid chromatography (HPLC, Figure A1). Extracts from* G. chinensis* have antibacterial activities against* S. aureus, Escherichia coli*, and* Streptococcus mutans,* including growth-inhibitory and biofilm-reducing effects [9, 13].

According to previous reports, the exposure of specific pathogen free mice to* G. chinensis* extract at 40 mg/L is unlikely to result in significant toxicity [33]. For the antimicrobial susceptibility testing of* S. aureus* biofilm, our results showed that biofilms of* S. aureus* treated with different concentrations of* G. chinensis* extract solutions were significantly impaired when compared with the untreated group. The MIC value of MRSA for* G. chinensis* aqueous extract was 31.25 *μ*g/mL, which is within the range of what is known to be safe. When treated with 1/2MIC of* G. chinensis* extract, the growth of the planktonic* S. aureus* strain was consistently inhibited and had a turbid bacterial suspension appearance (Figures A2 and A3).

The staphylococcal biofilm substance consists of polysaccharide intercellular adhesion (PIA), protein, and extracellular DNA (eDNA), which provides strength to the biofilm [34]. The present results indicated that the water extract of* G. chinensis* effectively inhibited the production of the extracellular substance matrix during biofilm formation. Additionally, our findings from CLSM showed that the water extracts of* G. chinensis* could inhibit the EPS/bacteria ratio in the biofilm aggregation on the bone specimens, which was probably related to the bactericidal effect against* S. aureus*. From this observation, we inferred that* G. chinensis* extract led to the downregulation of genes involved in biofilm formation and exopolysaccharide synthesis.

Interestingly, transcriptome analysis suggested that* G. chinensis* extract downregulated the expression of methicillin-resistant* S. aureus* biofilm-associated genes and exopolysaccharide synthesis genes. These findings are crucial since carbohydrate metabolism and exopolysaccharide synthesis are the key virulence factors for the biofilm formation of MRSA strains [5]. Transcriptome analysis confirmed the decrease in carbohydrate metabolism in the* G. chinensis* extract-treated methicillin-resistant* S. aureus* along with a reduced exopolysaccharide matrix in the biofilms. The YycFG TCS plays an essential role in cellular physiology, structure, and biofilm organization, particularly in cell wall metabolism [35]. The matrix of the three-dimensional staphylococcal biofilm is mainly composed of PIAs encoded by* the ica *operon (glycosyl transferase family protein) in* S. aureus* [36].

Osteomyelitis is a common disease of a major challenge for clinical treatment, particularly when infected by methicillin-resistant* S. aureus*, which often requires a combination of aggressive surgery and extended antibiotic therapy [37]. In this study, we validated the role of the* G. chinensis* extract in limiting the invasive ability and pathogenicity of methicillin-resistant* S. aureus in vivo*. We recorded reactive bone formation and bone infarction surrounding the bone tissues infected by MRSA strains in a rat model. The results indicated that* G. chinensis* extract inhibited the invasive ability and pathogenicity of MRSA* in vivo*. However, the limitation of the present study was a lack of the histology methods to observe, osteoblast, and osteoclast which would be considered in the future.

## 5. Conclusions

In summary, the results indicated the sensitivity of methicillin-resistant* S. aureus* to the* G. chinensis* water extracts. Furthermore, we showed that* G. chinensis* extract leads to a reduction in dextran-dependent aggregation and biofilm formation in* S. aureus* biofilms. Based on the transcriptome analysis,* G. chinensis* extract significantly affected the expression of several genes related to biofilm formation and influenced carbohydrate metabolism in methicillin-resistant* S. aureus*. Furthermore, we showed that* G. chinensis* extract inhibited the invasive ability and pathogenicity of methicillin-resistant* S. aureus in vivo*. Taken together, the antimicrobial properties of* G. chinensis* extract are probably linked to its modulation of methicillin-resistant* S. aureus* carbohydrate metabolism, which makes it a potential compound useful for the management of methicillin-resistant* S. aureus *infections.

## Data Availability

The data used to support the findings of this study are included within the article.

## List of Abbreviations

*S. aureus*: * Staphylococcus aureus*
*G. chinensis*: * Galla chinensis*
qRT-PCR: Quantitative real-time PCR
MIC: Minimum inhibitory concentration
SEM: Scanning electron microscopy
CLSM: Confocal laser scanning microscopy
RNA-seq: RNA sequencing
HPLC: High performance liquid chromatography.

## Supplementary Materials

Supplementary Materials Table A1: sequences of primers used for qRT-PCR analysis. Table A2: the minimum inhibitory concentration values for MRSA strain. Figure A1: culture on the solid medium and Gram's staining for* S. aureus*. Figure A2: chemical fingerprint of* Galla chinensis* by HPLC. Figure A3: planktonic* S. aureus* ATCC29213 treated with different concentrations of* G. chinensis* extract solutions. Figure A4: planktonic MRSA strains treated with different concentrations of* G. chinensis* extract solutions. Figure A5: the histology methods for the evaluation of the infective tibias in rats.
