# Supplementary material for: The Pathogenicity and Transcriptome Analysis of Methicillin-Resistant Staphylococcus aureus in Response to Water Extract of Galla chinensis
Source: Evid Based Complement Alternat Med. 2019 Jul 16;2019:3276156. doi: 10.1155/2019/3276156 (PMC6662456; doi:10.1155/2019/3276156)
Supplement: Supplementary Materials — Table A1: sequences of primers used for qRT-PCR analysis. Table A2: the minimum inhibitory concentration values for MRSA strain. Figure A1: culture on the solid medium and Gram's staining for S. aureus. Figure A2: chemical fingerprint of Galla chinensis by HPLC. Figure A3: planktonic S. aureus ATCC29213 treated with different concentrations of G. chinensis extract solutions. Figure A4: planktonic MRSA strains treated with different concentrations of G. chinensis extract solutions. Figure A5: the histology methods for the evaluation of the infective tibias in rats. [file 3276156.f1.docx]

| **Appendices**  **Table A1 Sequences of primers used for qRT-PCR analysis** | | |
| --- | --- | --- |
| **Primers** | **sequence 5’-3’ (Forward/Reverse)** | **Reference** |
| **RT-qPCR** |  |  |
| *icaA* | 5’- GATTATGTAATGTGCTTGGA -3’/  5’- ACTACTGCTGCGTTAATAAT - 3’ | This study |
| *icaD* | 5’- ATGGTCAAGCCCAGACAGAG -3’/  5’- CGTGTTTTCAACATTTAATGCAA -3’ | This study |
| *icaB* | 5’- CACATACCCACGATTTGCAT -3’/  5’- TCGGAGTGACTGCTTTTTCC -3’ | This study |
| *yycF* | 5’ - TGGCGAAAGAAGACATCA -3’/  5’ – AACCCGTTACAAATCCTG- 3’ | This study |
| *yycG* | 5’ - CGGGGCGTTCAAAAGACTTT -3’/  5’ - TCTGAACCTTTGAACACACGT -3’ | This study |
| *yycH* | 5’ - TCAGTCAGGCGAGCTAACAT -3’/  5’ –CGCTAAGCTTGAACGTACAGA -3’ | This study |
| *16S rRNA* | 5’ - GTAGGTGGCAAGCGTTATCC -3’/  5’ –CGCACATCAGCGTCAACA-3’ | This study |
|  |  |  |

**Table A2. The minimum inhibitory concentration values for MRSA strain**

|  | *G. chinensis* | Vancomycin |
| --- | --- | --- |
| MIC (μg/mL) | 31.25 | 2 |


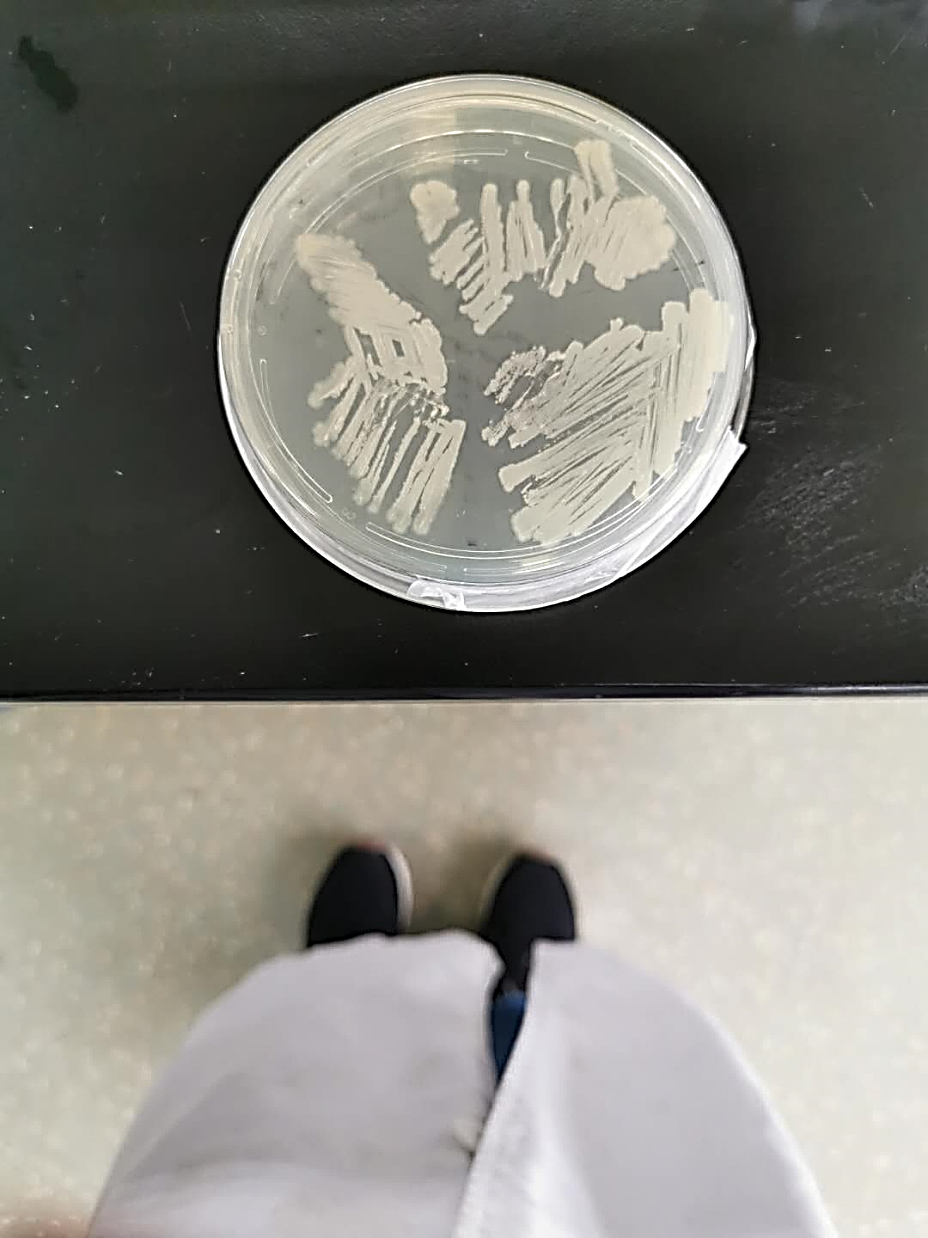

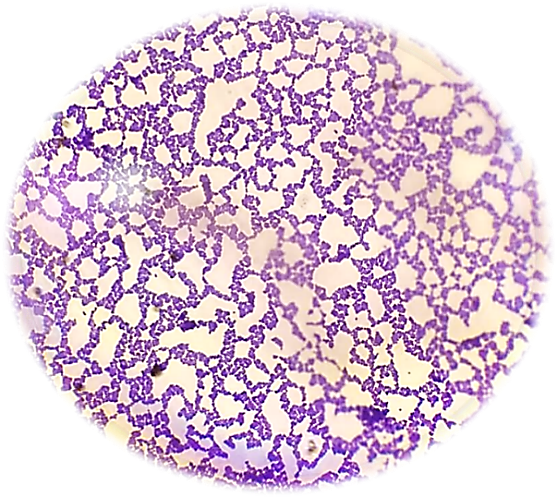


**Culture on BP agar plate Gram’s staining**

**Figure A1 Culture on the solid medium and Gram’s staining for *S. aureus***


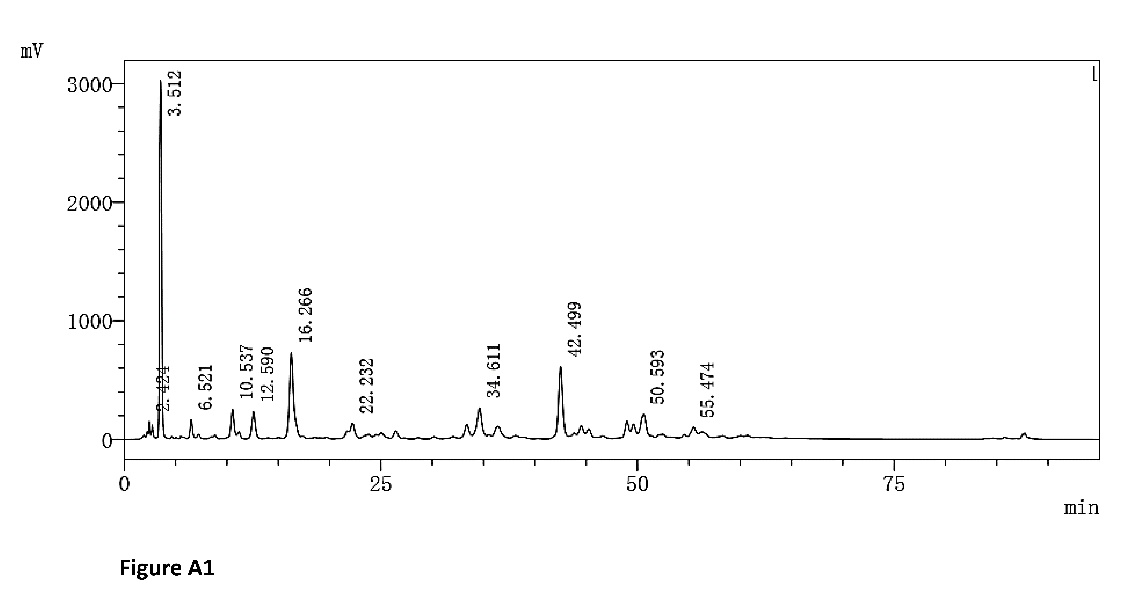


**Figure A2** Chemical fingerprint of *Galla Chinensis* by HPLC


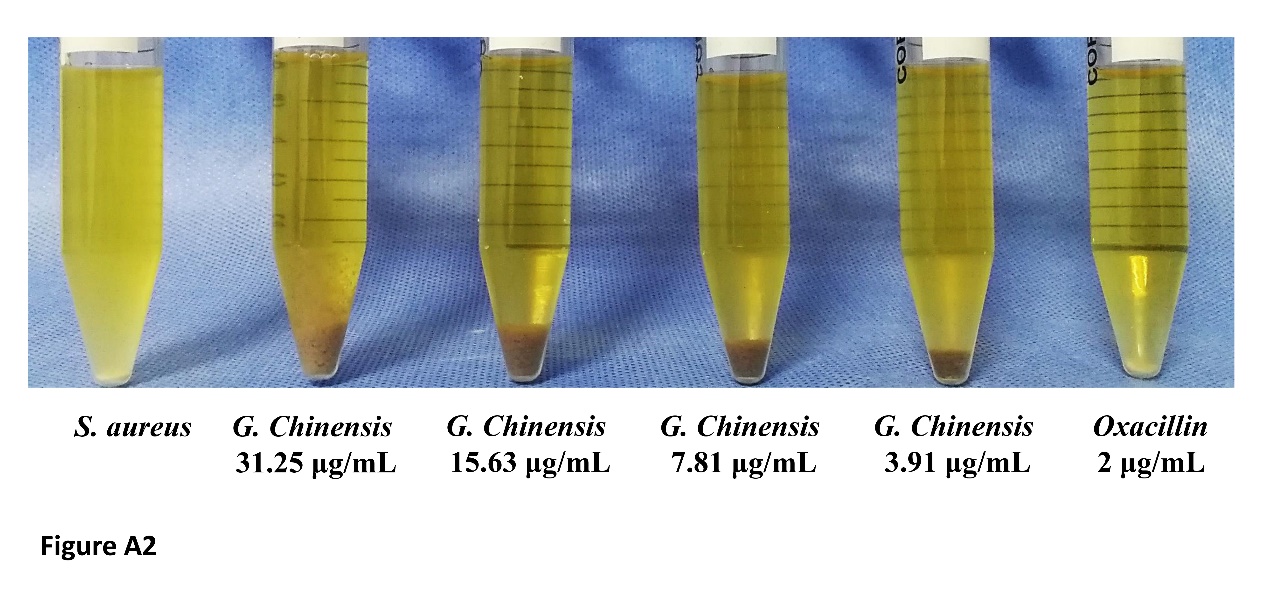


**Figure A3** Planktonic *S. aureus* ATCC29213 treated with different concentrations of *G. Chinensis* extract solutions


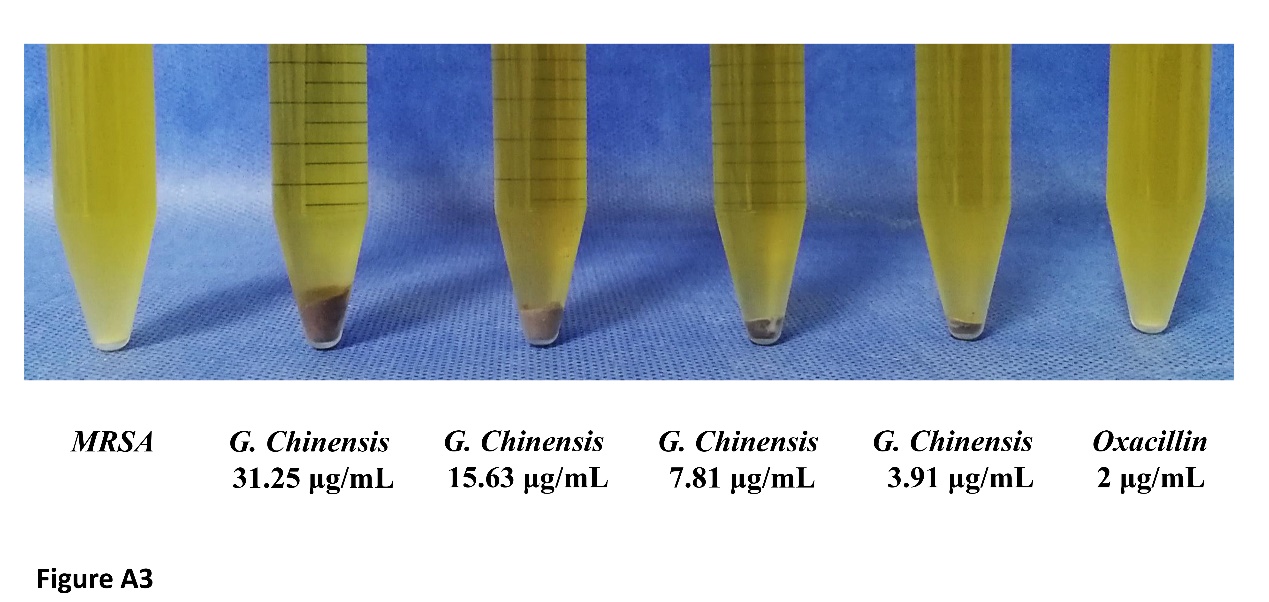


**Figure A4** Planktonic MRSA strains treated with different concentrations of *G. Chinensis* extract solutions

**
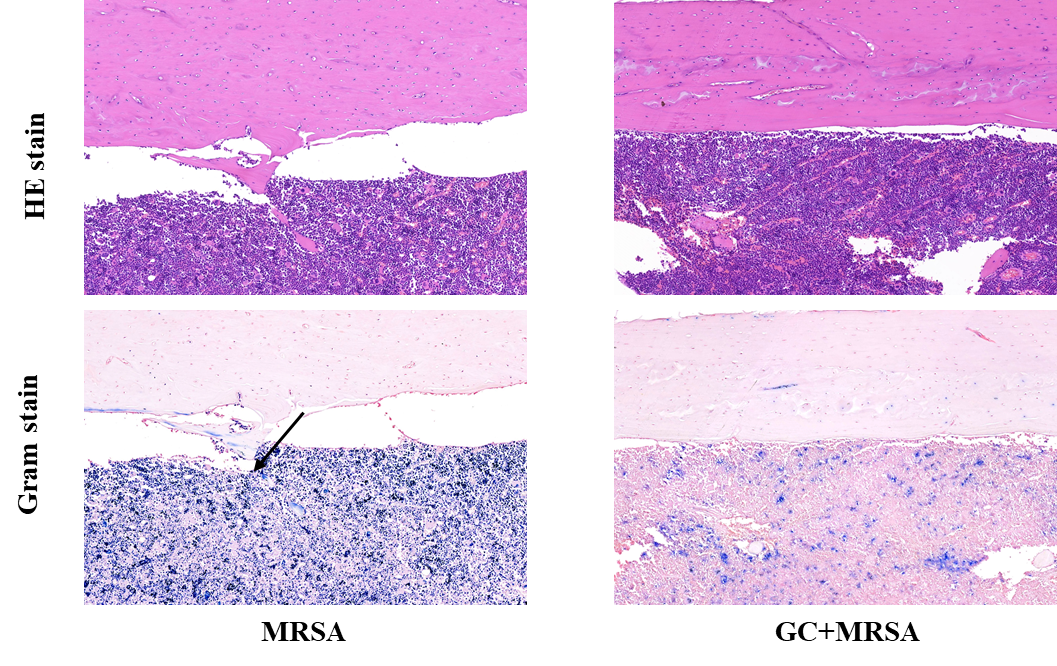
**

**Figure A5 The histology methods for the evaluation of the infective tibias in rats**

Black arrow indicated the Gram-positive cells
